# A comparative study of digital PCR and real-time qPCR for the detection and quantification of HPV mRNA in sentinel lymph nodes of cervical cancer patients

**DOI:** 10.1186/s13104-017-2846-8

**Published:** 2017-10-30

**Authors:** Katrin Carow, Christina Read, Norman Häfner, Ingo B. Runnebaum, Adam Corner, Matthias Dürst

**Affiliations:** 1 Klinik und Poliklinik für Frauenheilkunde und Fortpflanzungsmedizin, Universitätsklinikum Jena, Am Klinikum 1, 07747 Jena, Germany; 2 RainDance Technologies, 749 Middlesex Turnpike, Billerica, MA 01821 USA

**Keywords:** HPV mRNA, Molecular marker, Sentinel lymph node, Cervical cancer, Recurrence, Quantitative PCR, Digital PCR

## Abstract

**Background:**

Qualitative analyses showed that the presence of HPV mRNA in sentinel lymph nodes of cervical cancer patients with pN0 status is associated with significantly decreased recurrence free survival. To further address the clinical potential of the strategy and to define prognostic threshold levels it is necessary to use a quantitative assay. Here, we compare two methods of quantification: digital PCR and standard quantitative PCR.

**Methods:**

Serial dilutions of 5 ng–5 pg RNA (≙ 500–0.5 cells) of the cervical cancer cell line SiHa were prepared in 5 µg RNA of the HPV-negative human keratinocyte cell line HaCaT. Clinical samples consisted of 10 sentinel lymph nodes with varying HPV transcript levels. Reverse transcription of total RNA (5 µg RNA each) was performed in 100 µl and cDNA aliquots were analyzed by qPCR and dPCR. Digital PCR was run in the RainDrop^®^ Digital PCR system (RainDance Technologies) using a probe-based detection of HPV E6/E7 cDNA PCR products with 11 µl template. qPCR was done using a Rotor Gene Q 5plex HRM (Qiagen) amplifying HPV E6/E7 cDNA in a SYBR Green format with 1 µl template.

**Results:**

For the analysis of both, clinical samples and serial dilution samples, dPCR and qPCR showed comparable sensitivity. With regard to reproducibility, both methods differed considerably, especially for low template samples. Here, we found with qPCR a mean variation coefficient of 126% whereas dPCR enabled a significantly lower mean variation coefficient of 40% (p = 0.01). Generally, we saw with dPCR a substantial reduction of subsampling errors, which most likely reflects the large cDNA amounts available for analysis.

**Conclusions:**

Compared to real-time PCR, dPCR shows higher reliability. Thus, our HPV mRNA dPCR assay holds promise for the clinical evaluation of occult tumor cells in histologically tumor-free lymph nodes in future studies.

**Electronic supplementary material:**

The online version of this article (10.1186/s13104-017-2846-8) contains supplementary material, which is available to authorized users.

## Background

In patients with cervical cancer, metastatic spread to lymph nodes is known to be the most significant prognostic parameter. However, up to 15% of patients with no evidence of lymph node metastasis (pN0) develop recurrent disease [1, 2]. This may be due to occult metastatic spread of tumor cells. In a recent study we used human papillomavirus (HPV) mRNA as a highly specific molecular marker for disseminated tumor cells to predict the risk of recurrence. Indeed, in patients with cervical cancer and pN0-status (assessed by conventional histopathology; no ultra-staging and no immunohistochemistry) the presence of HPV mRNA in sentinel lymph nodes (SLN) was found to be of prognostic value independent of tumor size [3]. A weakness of that study was that HPV mRNA detection was based on a nested-PCR approach which is extremely sensitive but only allows a qualitative read out. For clinical implementation as well as for the definition of a prognostic threshold level for HPV mRNA, it is important to extend analyses with a quantitative assay. The current gold standard for quantification is quantitative real-time PCR (qPCR) [4]. However, qPCR has limited applicability due to the high standard deviations typically observed with low template numbers. The analysis of larger amounts of cDNA to improve standard deviation is no alternative, since high amounts of non-template cDNA would inhibit the reaction. An alternative approach is digital PCR (dPCR) [5]. This technique allows a reduction of background per template molecule due to reaction partitioning into droplets and enables in this way the analysis of large cDNA amounts. Here, we compare both methods by detecting single template molecules within a large background of non-specific cDNA. Our aim was to determine which technique provides the most accurate, sensitive, and reproducible results for HPV mRNA quantification in SLN of cervical cancer patients.

## Materials and methods

### Samples and sample preparation

RNA was harvested from two cell lines: SiHa cells (HPV16 positive cervical carcinoma cell line; ATCC^®^, Catalog Number HTB-35™) and HaCaT cells (HPV-negative immortalized human keratinocyte cell line; CLS Cell lines service, Catalog Number 300493). A tenfold serial dilution of 5 ng–5 pg of RNA from SiHa cells (≙ 500–0.5 cells) was prepared in a background of 5 µg of HaCaT RNA. Additionally, RNA from SLN biopsies was isolated. Because of the explorative nature of this study which did not intend to test a pre-planned confirmatory hypothesis, a statistically justified sample size was not calculated. Instead, a small sample set was chosen which focused on preselected SLN biopsies. Of 10 samples, 7 samples were previously identified as having low HPV16 transcript levels (< 5 transcript equivalents/50 ng RNA). The remaining 3 samples had high transcript levels (> 100 transcript equivalents/50 ng RNA) [6]. Approximately 30 mg of tissue from each SLN were homogenized and total RNA was extracted using the RNA Blood Mini Kit (Qiagen, Catalog Number 52304). RNA concentration was determined by NanoDrop.

For cDNA synthesis of clinical samples as well as for the serial dilution series, 5 µg of total RNA were reverse transcribed in a 80 μl reaction comprising 40 pmol CDS-primer (5′-T_n= 30_VN-3′), 40 pmol of each dNTP, first strand buffer, 5 mM DTT, 80 U RNaseOUT™ (Thermo Fisher Scientific, Catalog Number 10777019) and 400 U SuperScriptII (Thermo Fisher Scientific, Catalog Number 18064014). To allow optimal annealing, primer and RNA were incubated at 65 °C for 5 min and cooled down on ice. The remaining reagents were then added in form of a master mix and cDNA synthesis was done at 42 °C for 1 h. The reaction was stopped at 70 °C for 15 min, adjusted to 100 µl with *aqua dest.* and stored in aliquots at − 80 °C until PCR amplification. To rule out contamination we performed isolation and reverse transcription of HPV-negative samples in a non-HPV laboratory.

### qPCR and dPCR

Both, qPCR and dPCR, were performed with assays designed to detect all HPV16 mRNA transcripts (cDNA) initiated at promoter p97 irrespective of splicing (Table 1).

**Table 1:** Oligonucleotides used for amplification with qPCR and dPCR

Oligonucleotide	Sequence (5′ → 3′)	HPV position
qPCR forward primer	AATGTTTCAGGACCCACAGG	103–122
qPCR reverse primer	CTCACGTCGCAGTAACTGTTG	206–226
dPCR forward primer	TTCGGTTGTGCGTACAAAGC	755–774
dPCR reverse Primer	AGTGTGCCCATTAACAGGTCTTC	799–821
dPCR TaqMan-MGB probe	CACGTAGACATTCGTACTT	778–796

qPCR was done using 1 µl cDNA in a Rotor Gene Q 5plex HRM (Qiagen) in triplicate runs each performed with triplicate reactions (9 × reactions in total). The 25 µl reaction consisted of 6 pmol of each dNTP, 10 pmol of forward and reverse primer, respectively, 5% DMSO, 1.75 mM MgCl_2_, 10 mM Tris–HCl pH 8.3, 50 mM KCl, 0.001% gelatine and AmpliTaqGold (1.25 U) (Applied Biosystems, Catalog Number N8080247). Cycling included a 10 min initial denaturation step at 95 °C, 45 cycles of 15 s denaturation (95 °C), 20 s annealing (58 °C) and 30 s elongation (72 °C) and 4 min final elongation step (72 °C). The melting temperature of the PCR product was determined to ensure specificity. A serial dilution of 1 pg–10^−4^ pg (≙ 100,000–10 copies) of plasmid-cloned HPV16 genome served as quantification standard. A further dilution step of the standard was excluded due to unreliable quantification results with only one plasmid copy per reaction. Four no template controls (NTC) were included in each qPCR run. Triplicate runs of dPCR were done on a RainDrop Digital PCR System (RainDance Technologies) with single replicates of 11 µl cDNA. dPCR was performed in a 50 µl reaction with Genotyping Master Mix (Thermo Fisher Scientific, Catalog Number 4371355), 45 pmol of forward and reverse primer, respectively, and 10 pmol of TaqMan probe (Table 1). Cycling included 10 min initial denaturation (95 °C), 45 cycles of 15 s denaturation (95 °C) and 60 s annealing (61 °C) followed by 10 min at 98 °C and finally 10 min at 12 °C. Each run contained 2 NTCs. Only validated reagents were used throughout the study; PCR primers and probes were synthesized by Eurofins Genomics.

Standard deviation and detection limits were calculated and compared for both methods. Calculations on statistical significance were performed with two-tailed t test after ensuring standard normal distribution by Kolmogorov–Smirnov test.

## Results

**Fig. 1:**
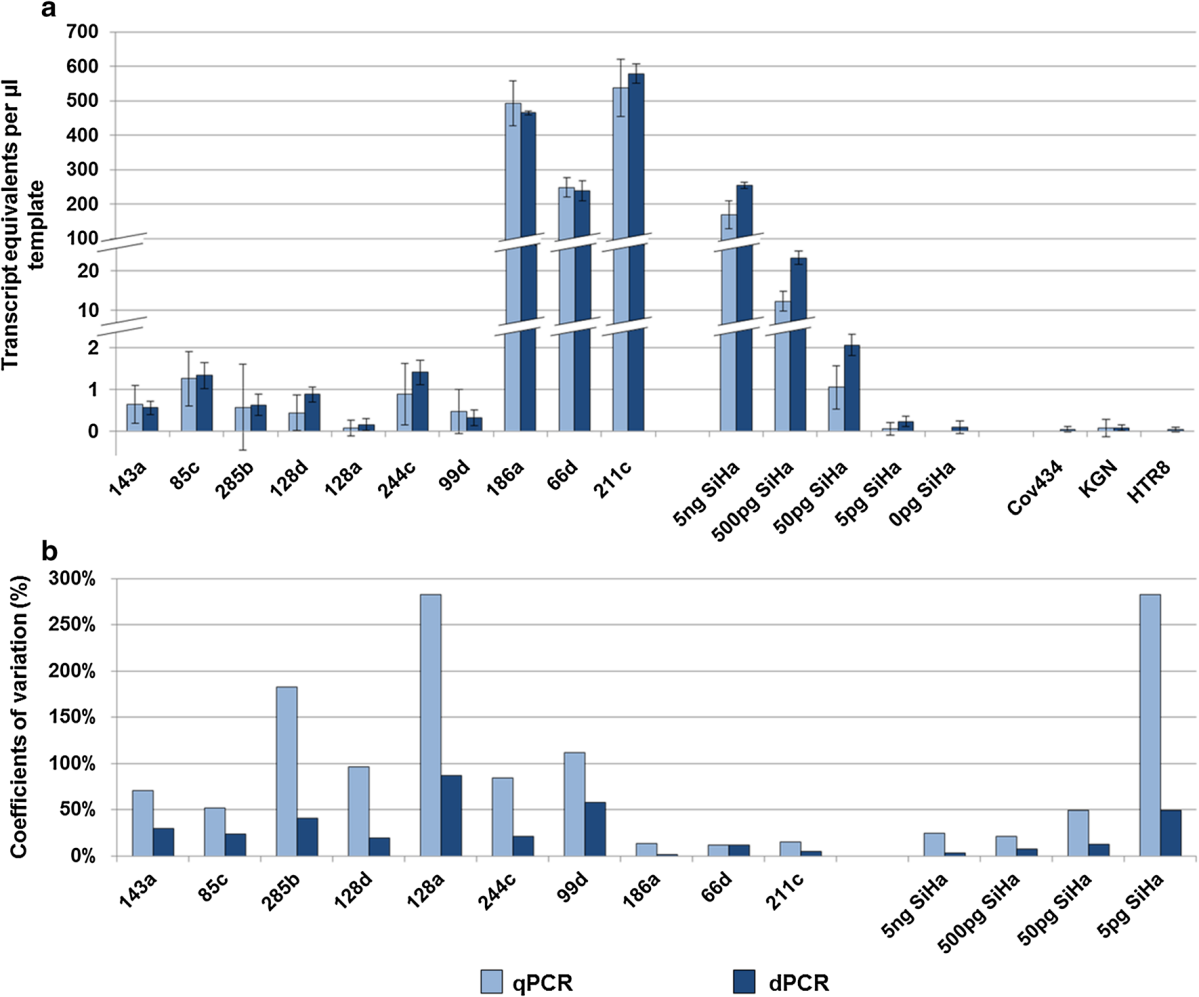
Comparision of qPCR and dPCR. **a** Results of HPV transcript quantification by both methods. Calculated transcript equivalents per µl template (y-axis) are shown for 10 sentinel lymph nodes, a dilution series of SiHa cells and 3 HPV-negative control cell lines (x-axis). Standard deviations are indicated by error bars. **b** Detection reliability of the above samples by calculation of coefficients of variations. The coefficients of variation are considerably lower in dPCR analyses. This benefit is especially obvious in samples with a low number of templates

We tested two methods for quantification of HPV16 mRNA in SLN of cervical cancer patients: our standard qPCR and a newly established dPCR approach. Both techniques showed comparable results for clinical samples as well as a serial dilution series of RNA from a HPV16 positive cell line (SiHa) in RNA from a HPV negative cell line (HaCaT) (Fig. 1a; see Additional file 1: Raw data).

Detection reliability was evaluated by calculation of coefficients of variation from standard deviations. Both methods displayed equal analytical sensitivity and reproducibility, which were determined by assessing the respective parameter with regard to “copies per reaction”: in dPCR, 11 µl of the 5 pg SiHa sample contained transcript equivalents of 0.55 target cells per reaction and resulted in 100% positive replicates with a CV of 50%. The comparable qPCR pendant is 1 µl of the 50 pg SiHa step with 0.5 target cells per reaction. This was likewise detected with 100% positive replicates and a CV of 53% (Fig. 1b).

The study was designed to examine the suitability of each method for analyzing low template samples. Consequently we next focused on a sample specific perspective rather than on an analytical mode of interpretation. The highest dilution step comprising 5 pg SiHa RNA in a 100 µl cDNA synthesis reaction (0.005 target cell equivalents per microliter) was detected by both techniques. dPCR analyses resulted in 3/3 positive replicates with approximately 0.55 pg template (11 µl template ≙ 0.055 target cells) per reaction. Not surprisingly, the qPCR analyses were limited by stochastic effects resulting from the low input volume (1 µl) and achieved 1/9 positive replicates with approximately 0.05 pg template (≙ 0.005 cells) per reaction. This effect is even more evident by calculating variation coefficients: throughout the dilution series we obtained CVs between 3.5 and 49% when using dPCR, whereas qPCR resulted in CVs between 24 and 282%. In high template clinical samples, dPCR yielded a mean CV of 6%. In comparison, qPCR yielded a mean CV of 13%. This difference is not significant. In contrast, by analyzing low template samples we found a significant difference with a mean CV of 40% for dPCR and a mean CV of 126% for qPCR (p = 0.01).

Of note is that the results of low template samples are challenged by the presence of unspecific signals in both techniques. For dPCR, these are related to a background signal (1 copy per 55,000 cells) which is evident in the HaCaT only dilution step, in all 3 control cell lines (Cov434, KGN and HTR8) comprising HPV negative cDNA and in one of six NTCs. In qPCR a contamination was observed in one of nine reactions for the KGN control whereas all other controls remained negative.

## Discussion

In this comparative study, we examined the suitability of dPCR and qPCR for quantifying HPV mRNA in SLN of cervical cancer patients. The performance of both techniques was assessed by analyzing RNA from SLN biopsies and a serial dilution of RNA derived from HPV16 positive SiHa cells in a background of HPV negative HaCaT RNA. To test the applicability over a broad dynamic range and with challenging specimens, samples were selected to display low as well as high target abundance.

Overall absolute sensitivity was similar but dPCR showed a superior performance with regard to reproducibility. These results are in concordance with observations made elsewhere [7, 8] and are particularly relevant for samples with low target concentration. The improvement is related to larger input volumes in dPCR. To use higher amounts of template for qPCR in order to compensate for the larger volume used for dPCR is no alternative since our analyte already had a high concentration (50 ng RNA per µl in cDNA synthesis). Higher amounts of nucleic acids would simply hamper qPCR. In contrast, dPCR enables a partitioning of the background leading to a reduction of the inhibiting effect of excess cDNA as well as to a reduction of the background per template molecule. The latter is considered as one main advantage of dPCR [9].

Digital PCR has been used for viral nucleic acid quantification in numerous studies [7, 10–12]. However, only Biron and colleagues applied the technique for HPV detection and demonstrated the proof-of-principle of the strategy by HPV16 E6 E7 RNA quantification in oropharyngeal squamous cell carcinoma [12]. The study lacks the direct comparison of qPCR and dPCR, which we provide here in the context of cervical carcinoma.

Both techniques are challenged by the presence of false positive controls. The occurrence of false positive droplets is a well-known phenomenon of dPCR [7, 13]. Of note is that we never observed more one false positive droplet per reaction. This would be indicative of one HPV template per 55,000 cells. We can exclude sample contamination because all HPV-negative samples were processed (cell culturing, RNA extraction and cDNA synthesis) in a non-HPV laboratory. Further optimization of the TaqMan probe might solve this problem. For qPCR only one of 60 control reactions (comprising HPV negative cell lines and NTC) was false positive demonstrating that despite all precautions occasional low copy contamination is not always avoidable. Clearly, this needs to be considered in future studies when defining a clinically relevant threshold for predicting recurrence.

## Conclusion

The sensitivity and simplicity of qPCR is undisputable. However, dPCR showed a superior performance with regard to template input amounts and reproducibility. Together these points indicate a high potential for dPCR for the evaluation of the clinical relevance of occult tumour cells in histologically tumour-free lymph nodes of cervical cancer patients in future studies.

## Additional file

**Additional file 1.** Raw data generated by qPCR and dPCR. Ct values of 3 individual qPCR and dPCR runs respectively are shown for clinical samples and cell lines. A plasmid standard dilution series was done for qPCR only and the respective Ct values are shown. Calculated mean values, standard deviations and variation of coefficient are shown for both qPCR and dPCR.

## Abbreviations

Ct: cycle threshold
CV: coefficient of variation
dPCR: digital PCR
HPV: human papilloma virus
NTC: no template control
SCC: squamous cell carcinoma
SLN: sentinel lymph node
qPCR: quantitative PCR
pN0: no lymph node metastasis by histopathology
